# Endothelial nitric oxide synthase gene polymorphism (Glu298Asp) and development of pre-eclampsia: a case-control study and a meta-analysis

**DOI:** 10.1186/1471-2393-6-7

**Published:** 2006-03-16

**Authors:** Christina KH Yu, Juan P Casas, Makrina D Savvidou, Manpreet K Sahemey, Kypros H Nicolaides, Aroon D Hingorani

**Affiliations:** 1 Harris Birthright Research Centre for Fetal Medicine, King's College, Hospital, Medical School, Denmark Hill, London, SE5 9RS, UK; 2 Centre for Clinical Pharmacology, Department of Medicine, BHF Laboratories at UCL, Rayne Building, 5 University Street, London WC1E 6JJ, UK; 3 Department of Epidemiology and Population Health, London School of Hygiene and Tropical Medicine, Keppel Street, London WC1E 7HT, UK

## Abstract

**Background:**

Pre-eclampsia is thought to have an important genetic component. Recently, pre-eclampsia has been associated in some studies with carriage of a common eNOS gene Glu298Asp polymorphism, a variant that leads to the replacement of glutamic acid by aspartic acid at codon 298.

**Method:**

Healthy women with singleton pregnancies were recruited from 7 district general hospitals in London, UK. Women at high risk of pre-eclampsia were screened by uterine artery Doppler velocimetry at 22–24 weeks of gestation and maternal blood was obtained to genotype the eNOS Glu298Asp polymorphism. Odds ratios (OR) and 95%CI, using logistic regression methods, were obtained to evaluate the association between the Glu298Asp polymorphism and pre-eclampsia. A meta-analysis was then undertaken of all published studies up to November 2005 examining the association of eNOS Glu298Asp genotype and pre-eclampsia.

**Results:**

89 women with pre-eclampsia and 349 controls were included in the new study. The Glu298Asp polymorphism in a recessive model was not significantly associated with pre-eclampsia (adjusted-OR: 0.83 [95%CI: 0.30–2.25]; p = 0.7). In the meta-analysis, under a recessive genetic model (1129 cases & 2384 controls) women homozygous for the Asp298 allele were not at significantly increased risk of pre-eclampsia (OR: 1.28 [95%CI: 0.76–2.16]; p = 0.34). A dominant model (1334 cases & 2894 controls) was associated with no increase of risk of pre-eclampsia for women carriers of the Asp298 allele (OR: 1.12 [95%CI: 0.84–1.49]; p = 0.42).

**Conclusion:**

From the data currently available, the eNOS Glu298Asp polymorphism is not associated with a significant increased risk of pre-eclampsia. However, published studies have been underpowered, much larger studies are needed to confirm or refute a realistic genotypic risk of disease, but which might contribute to many cases of pre-eclampsia in the population.

## Background

Normal pregnancy is associated with a substantial change in maternal cardiovascular haemodynamics. Despite an increase in blood volume and cardiac output, blood pressure falls in the first half of pregnancy, as a result of systemic arteriolar vasodilatation, before returning to pre-pregnancy levels towards term [1]. Enhanced synthesis of the endothelium-derived mediator nitric oxide (NO), from L-arginine by endothelial nitric oxide synthase (eNOS), plays an important role in this vasodilatation [2,3]. Although the role of NO in the pathogenesis of pre-eclampsia is much debated, many studies support the association of reduced NO availability in hypertensive disorders of pregnancy [4,5].

Pre-eclampsia is thought to have an important genetic component [6,7]. Several studies have reported associations between pre-eclampsia and polymorphisms of various genes [8-10]. However, the lack of reproducibility of genetic case-control studies has led to uncertainty about the nature and number of genes contributing to pre-eclampsia risk [11]. Recently, pre-eclampsia has been associated with carriage of a common eNOS gene Glu298Asp polymorphism, a variant that leads to the replacement of glutamic acid by aspartic acid at codon 298. This variant is associated with reduced endothelium-dependent vasodilatation in pregnancy [12] and has also been associated with risk of ischaemic heart disease [13] for which pre-eclampsia is also a risk factor [14]. However conflicting results, mainly derived from individually underpowered studies, have been reported [15-23].

We have undertaken a new, case-control study of the eNOS Glu298Asp polymorphism and pre-eclampsia and combined our results with all available studies evaluating such an association in a meta-analysis.

## Methods

### Study population

Maternal genotyping was performed in women who underwent screening by uterine artery Doppler velocimetry at 22–24 weeks of gestation. This sub-study was part of a multi-centre randomised controlled trial that evaluated aspirin vs placebo as an intervention for the prevention of pre-eclampsia and the rationale and design of the study has been described in detail elsewhere [24]. Briefly, the study was conducted between January 2001 and July 2002 in seven hospitals in London, UK. Transvaginal Doppler examination of the uterine arteries was performed in 19,950 women with singleton pregnancies attending for routine ultrasound examination at 22–24 weeks of gestation, as previously described [25]. Women with pre-existing hypertensive, cardiovascular or renal diseases, diabetes mellitus, bleeding disorders or systemic lupus erythematosus, multiple pregnancies and pregnancies complicated by fetal abnormalities were excluded.

For the present study 623 women who had a mean pulsatility index (PI) of the uterine arteries above the 95^th ^centile (1.6) and 650 women with a mean PI <1.6, that consented to give a blood sample were followed up to delivery. Of the 1273 women, DNA was available for analysis in 89 women of the 121 who developed pre-eclampsia. Baseline characteristics of the 32 women that were not included in the DNA analysis for logistical reasons were not significantly different from the 89 cases included in the present report (Data not shown). Of the 1152 women who did not develop pre-eclampsia, 349 women (ratio 1:4) were selected as controls using frequency matching by maternal age and ethnic background.

Pre-eclampsia was defined according to the International Society for the Study of Hypertension in Pregnancy [26]. It required two recordings of diastolic blood pressure of 90 mmHg or higher at least fours hours apart or one recording of diastolic blood pressure of at least 120 mmHg or higher, in previously normotensive women, and urine protein excretion of at least 300 mg in 24 hours or two readings of 2+ or higher on dipstick analysis of midstream or catheter urine specimens if no 24 hour collection was available. The study was approved by the Multi-Centre Research Ethics Committee as well as the local ethics committees of the individual hospitals. Written informed consent was obtained from all participants.

### DNA extraction and genotyping

DNA was extracted by means of the QIAamp blood minikit, the 894 G/T polymorphism in exon 7, which predicts a Glu298Asp amino acid substitution in the mature protein, was genotyped by polymerase chain reaction (PCR) with primer pairs 5'-CCCCTCCATCCCACCCAGTCAATCC-3' and 5'-AGGAAACGGTCGCTTCGACGTGCTG-3' and allele-specific restriction enzyme digestion. PCR was performed for 35 cycles in a volume of 30 μL. Denaturation was at 95°C, annealing at 63°C and a final extension at 72°C, all for 45 seconds. Ten microliters of each PCR product (151bp) was then subjected to restriction digestion with 2U Dpn II, which cuts only in the presence of T-allele at position 894 (corresponding to Asp298). Digested samples were resolved by electrophoresis. Genotyping was conducted in a blinded manner to the clinical status of the subjects included in the study.

### Statistical analysis

#### Case-control study

For the case-control study medians, proportions and their corresponding interquartile ranges (IQR) were used for descriptive purposes. To evaluate the presence of differences between groups unpaired Student's *t*, χ^2^, or Mann-Whitney tests were used as appropriate. Tests for Hardy-Weinberg equilibrium were performed by χ^2 ^analysis.

As an a priori hypothesis, the association between the eNOS Glu298Asp polymorphism and pre-eclampsia was evaluated under a recessive model of inheritance, based on previous results from the eNOS genotype on ischaemic heart disease risk [13]. However, additive and dominant models were also evaluated. Multivariate analysis using logistic regression methods was also conducted to control for potential confounders (maternal age, ethnic background and smoking status). For each odds ratio (OR) a P value and 95% confidence intervals (CI) were obtained.

#### Systematic review

Our results were then considered in the context of a meta-analysis. Two electronic databases (MEDLINE and EMBASE) were searched up to November 2005 for all studies evaluating the Glu298Asp polymorphism and pre-eclampsia in all languages. Terms used for the search were both MeSH terms and text words "endothelial nitric oxide synthase", "nitric oxide synthase", "pre-eclampsia", "pregnant hypertensive disorders" and "pregnancy hypertension" in combination with "genetic", "polymorphism(s)", "mutation", "genotype" or "gene(s)". Authors were contacted to obtain stratified data of genotype frequency by ethnic group, and disease severity (severe vs non-severe pre-eclampsia), and for information about the use of blinding of genotyping staff to case-control status. The search results were limited to "human". We searched for any additional studies in the references of all identified publications, and the option "related articles" in MEDLINE for all selected papers was also used. Two investigators independently reviewed the study's eligibility, and the inconsistencies were resolved by consensus. Women in whom pre-eclampsia developed on the background of existing hypertension were excluded from the present meta-analysis in order to improve the homogeneity of phenotype between studies. As in the case-control study, the prior hypothesis was that homozygosity for the Asp298 allele compared with carriers of Glu298 allele would be associated with an increased risk of pre-eclampsia. In subsidiary analyses the ORs for dominant and additive genetic models for the Glu298Asp polymorphism were also calculated. For the additive model, a per-allele OR of the rare allele (Asp298) was compared between cases and controls by assigning scores of 0, 1 and 2 to homozygotes for the common allele, heterozygotes, and homozygotes for the rare allele, respectively, and calculating ORs by logistic regression. Pooled ORs and 95%CI were calculated using random effect models by the method of the DerSimonian and Laird [27,28]. The DerSimonian and Laird *Q *test [29] was used to evaluate the degree of heterogeneity between studies, and *I*^2 ^was used as a measure to describe the percentage of variability in point estimates that was due to heterogeneity rather than sampling error. A visual inspection of the Funnel plot, and the Egger regression asymmetry test were used to assess small-study bias, of which publication bias is one potential cause [30]. Stratified analysis by predefined characteristics, such as ethnicity, disease severity and use of blinding of genotyping staff to case-control status were conducted to investigate sources of heterogeneity [29].

Compliance to the Hardy-Weinberg equilibrium in the control samples from the studies included was also evaluated. Study populations were divided into Caucasians (White-British, White-Americans, Austrians and Finnish), Hispanics (American-Hispanics and Colombians), Afro-Caribbean (South Africans, British of Afro-Caribbean ancestors and Colombians of Afro-Caribbean ancestors) and Asians (Japanese, Koreans, Bangladeshi, and British of Indian ancestors), to analyse the effect of ethnicity on genotype frequencies and on genotype-disease association. A power calculation assuming that the frequency of the genotype at risk was 8% (as reported in Caucasians) under a recessive model of inheritance, indicated that to detect ORs of 1.3 a total of 2800 pre-eclamptic women and same number of controls respectively, would be required for 80% power at significance level of 0.05 (2-sided). Data were analysed using Stata 8.2 (Stata Corporation, College Station, Texas, 2004).

## Results

### Case-control study

No significant interaction between the use of prophylactic aspirin and the genotype-outcome relationship was observed (p = 0.85). As expected, women who developed pre-eclampsia had a higher body mass index (26.7 vs 25, p = 0.003) and increased mean uterine artery PI (1.85 vs 1.03, p<0.0001) compared with women who did not developed pre-eclampsia. Baseline characteristics of the 438 women (89 pre-eclamptic and 349 controls) according to the eNOS genotype are shown in Table 1. The distribution of the Glu298Asp genotypes in the 438 women analysed, did not differ significantly from that expected under the Hardy-Weinberg equilibrium (p = 0.14). No significant difference by ethnicity was observed among cases and controls (Caucasians: cases 51.7% and controls 57.9%; p = 0.35; Afro-Caribbean: cases 40.4 and controls 33.2%; p = 0.25; and other ethnic groups: cases 7.9% and controls 8.9%; p = 0.93). There was no significant difference in the genotype frequencies for Glu298Asp among cases (Glu/Glu: 60 [67%], Glu/Asp: 24 [27%] and Asp/Asp: 5 [6%]) and controls (Glu/Glu: 198 [67%], Glu/Asp: 125 [26%] and Asp/Asp: 26 [7%]). Under a recessive model of inheritance (Asp/Asp vs Glu/Asp plus Glu/Glu), no significant increase risk of pre-eclampsia was observed in a univariate (OR: 0.74 [95%CI: 0.28–1.98]; p = 0.65) or in a multivariate model (OR: 0.83 [95%CI: 0.30–2.25]; p = 0.70) after adjustment for potential confounders ethnic background, smoking and maternal age. Similar results were obtained for dominant and additive models of inheritance (Table 2 and 3).

**Table 1:** Baseline characteristics of 438 pregnant women evaluated according to eNOS genotype

**Characteristics**	**Glu/Glu (n = 258)**	**Glu/Asp (n = 149)**	**Asp/Asp (n = 31)**	P value*
Age in years, median (IQR)	30 (24–34)	30 (26–34)	30 (22–34)	0.36
Primiparous, n (%)	135 (52)	76 (51)	20 (65)	0.38
BMI, kg/m^2^; median(IQR)	25.1(22.3–28.8)	25.2 (17.3–29.4)	25.9 (23.6–28.8)	0.89
Smokers, n (%)	18 (7)	15 (10)	3 (10)	0.47
Mean pulsatility index median (IQR)	1.1 (0.96–1.54)	1.1 (0.9–1.36)	0.85 (0.6–1.11)	0.68

* Comparisons between the three genotype-groups for continuous variables by ANOVA test and for categorical variables by χ^2^test

IQR= interquartile ranges

Glu/Glu= Glutamic acid/Glutamic acid

Glu/Asp = Glutamic/Aspartate

Asp/Asp = Aspartate/Aspartate

BMI = body mass index

**Table 2:** Estimate of the effect of the eNOS Glu298Asp polymorphism on pre-eclampsia risk in the current case-control study stratified by ethnic group.

**Model of inheritance**	**Unadjusted OR (95%CI)**	***P***	Adjusted OR* (95%CI)	***P***
**Additive**				
***Asp/Asp vs Glu/Glu***				
All women	0.63 (0.23 – 1.72)	0.37	0.69 (0.25 – 1.92)	0.47
Caucasians	0.89 (0.30 – 2.59)	0.83	0.86 (0.29 – 2.52)	0.79
Afro-Caribbean	N.A		N.A	
Asians	N.A		N.A	
***Glu/Asp vs Glu/Glu***				
	0.64 (0.38 – 1.07)	0.08	0.64 (0.37 – 1.10)	0.10
Caucasians	0.77 (0.38 – 1.53)	0.46	0.78 (0.39 – 1.57)	0.50
Afro-Caribbean	0.49 (0.18 – 1.28)	0.14	0.49 (0.18 – 1.31)	0.15
Asians	0.86 (0.07 – 10.42)	0.91	0.80 (0.06 – 10.0)	0.86
				
**Recessive**				
***Asp/Asp vs (Glu/Asp+Glu/Glu)***				
All women	0.74 (0.28 – 1.98)	0.65	0.83 (0.30 – 2.25)	0.70
Caucasians	0.99 (0.35 – 2.79)	0.99	0.95 (0.34 – 2.69)	0.95
Afro-Caribbean	N.A		N.A	
Asians	N.A		N.A	
**Dominant**				
***(Asp/Asp+Glu/Asp) vs Glu/Glu***				
All women	0.63 (0.39 – 1.04)	0.07	0.65 (0.39 – 1.08)	0.09
Caucasians	0.79 (0.42 – 1.51)	0.49	0.80 (0.42 – 1.53)	0.51
Afro-Caribbean	0.46 (0.17 – 1.21)	0.11	0.45 (0.17 – 1.22)	0.11
Asians	0.52 (0.05 – 5.62)	0.59	0.51 (0.04 – 5.68)	0.58

*; ORs adjusted by maternal age, ethnic background and smoking status. In the comparisons of the eNOS genotype, the reference value for each comparison was set to 1.0. N.A; There were no women homozygous for the Asp-allele in the Afro-Caribbean or Asian ethnic group.

**Table 3:** Multiple regression adjusted odds ratio of pre-eclampsia.

**Variable**	**Risk Comparison**	**Odds ratio**	**95% Confidence interval**
eNOS/Glu298Asp	Asp/Asp vs Glu-carriers	0.83	0.30 – 2.25
Smoking	vs non-smokers	0.88	0.34 – 2.25
Maternal age	per 1 year increment	1.02	0.98 – 1.06
Ethnic group Caucasians		0.75	0.45 – 1.25
Asians	vs Afro-Caribbean	1.06	0.39 – 2.89

### Meta-analysis eNOS Glu298Asp and pre-eclampsia

Twelve genetic association studies (including the present study) of Glu298Asp polymorphism in pre-eclampsia involving 1334 cases and 2894 controls were included in the present meta-analysis (Table 4) [15-23,31,32]. Six of the twelve studies, were performed only in Asians [15,16,18,20,22,32], two only in Caucasians [17,21], one only in Afro-Caribbeans [31] and three studies (including the present study) conducted in a mixed population [19,23].

**Table 4:** Characteristics of published studies of the association between the eNOS genotype (Glu298Asp) and pre-eclampsia included in the meta-analysis

**Study-year (Country)**	**Study design**	**Matching**	Asp/Asp * genotype (%)	**Primiparous (%) Cases Controls**		**Proteinuria threshold**	**Systolic/Diastolic blood pressure threshold**	**Main exclusion criteria**
**Caucasians**								
Hakli T-2003 (Finland)	Case-control	Age	10.61	100	--	0.3 g/24 h	140/90	• Hypertension
Tempfer CB-2004‡ (Austria)	Case-control	Gestational age and parity	--	--	--	5 g/24 h	160/110	• Cardiac (Hypertension), diabetes mellitus or renal disease • Multiple pregnancies • Autoimmune disorders & history of recurrent miscarriage
**Oriental**								
Yoshimura T-2000 (Japan)	Case-control	Age	1.17	68.7	80	0.3 g/L	140/90	• Cardiac, diabetes mellitus or renal disease • Lupus erythematosus • Multiple pregnancies
Kobashi G-2001 (Japan)	Case-control	--	0	59.2	57	0.3 g/L	140/90	• Hypertension, diabetes mellitus or renal disease • Multiple pregnancies • Amniotic volume abnormalities or fetal anomalies
Watanabe H-2001‡ (Japan)	Case-control	--	3.12	--	--	2 g/24h	160/110	• Hypertension • Renal disease
Ohta K-2004‡ (Japan)	Case-control	--	0	100	100	2 g/24 h	160/110	• Hypertension, diabetes mellitus, renal disease • Multiple pregnancies • Amniotic volume abnormalities or fetal anomalies
**Kim YJ (Korea)**	Case-control	--	--	21	31	0.3 g/L	140/90	• Hypertension, diabetes mellitus or renal disease • Multiple pregnancies, recurrent miscarriages, fetal growth retardation or abruption placenta
**Asians**								
Yoshimura T-2003‡ (Bangladesh)	Case-control	Age	3.36	44.6	51.2	≥ 3+ dipstick	160/110	• Hypertension, Diabetes mellitus or renal disease • Lupus erythematosus • Multiple pregnancies
**Afro-Caribbeans**								
**Hillermann R‡ (South Africa)**	Case-control	--	1.19	100	--	0.3 g/L	--/90	• Hypertension, diabetes mellitus, renal disease
**Study-year (Country)**	**Study design**	**Matching**	Asp/Asp * genotype (%)	**Primiparous (%) Cases Controls**		**Proteinuria threshold**	**Systolic/Diastolic blood pressure threshold**	**Main exclusion criteria**

**Mixed population^†^**								
Landau R-2004 (United States)	Case-control	--	5.03	55	41	0.3 g/L	140/90	• Hypertension
Yu C. KH-2004 (United Kingdom)	Case-control	--	7.44	56.3	69.3	0.3 g/24 h	140/90	• Cardiac, diabetes mellitus or renal disease • Lupus erythematosus • Multiple pregnancies
Serrano NC-2004 (Colombia)	Case-control	--	1.14	100	100	0.3 g/24 h	140/90	• Cardiac (Hypertension), diabetes mellitus or renal disease • Multiple pregnancies • Lupus erythematosus

*; Genotype frequency from control subjects. ‡ Studies in which only women with severe pre-eclampsia were included as cases.

†; Landau R included Hispanic and White American women, Yu C.KH included Caucasian, Afro-Caribbean, and Asian women and Serrano N included Hispanic and Afro-Caribbean women.

The frequency in control samples of women homozygous for the Asp298 allele was overall 3.2% (95%CI: 0.6–5.7). However this frequency was different among ethnic groups as follows: Caucasians 9.5% (standard error [SE]: 1.61), Asians 1.41% (SE: 0.95), Hispanics 2.55% (SE: 1.53) and Afro-Caribbean 1.11% (SE: 0.71). All control samples from the studies included on which genotype frequency was available were in Hardy-Weinberg equilibrium (P>0.05) [15-20,22,23,31]. In two of the studies no homozygous women were detected among cases or controls [16,20] and in other study [21], the genotype frequency needed for analysis under a recessive model was not reported and the relevant information could not be obtained from the author. Therefore, we excluded these studies (205 cases & 510 controls) [16,20,21] for the analysis under a recessive and additive model but included them under a dominant model of inheritance. For the studies of Kobashi G, et al [16] and Ohta K, et al [20] a unified data-set of tabular data obtained from the authors was used to avoid duplication of subjects involved in the two studies.

**Figure 1:**
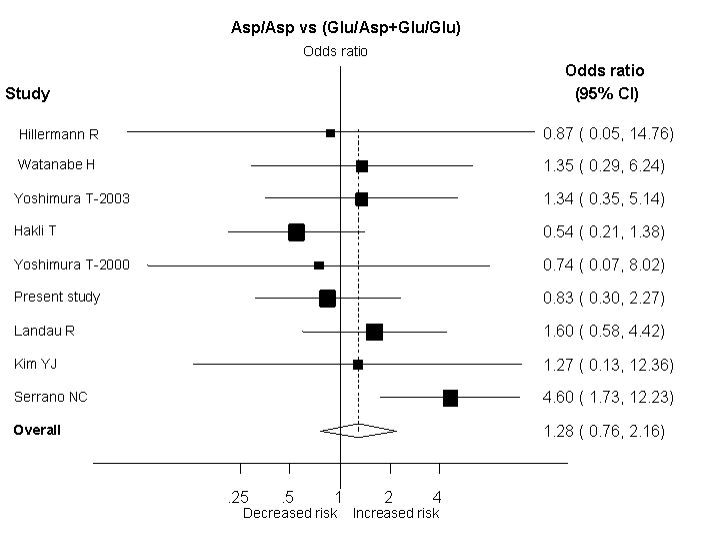
Results of published studies of the association between the eNOS Glu298Asp polymorphism and pre-eclampsia. ORs for the outcome compared homozygous women for the Asp298 allele vs heterozygous (Glu/Asp) plus wild type (Glu/Glu) women (Recessive model), sorted by study size.

After combining our results in a meta-analysis, the summary OR was 1.28 (95%CI: 0.76–2.16; p = 0.34) for women homozygous for the Asp298 allele compared with carriers of Glu298 allele (Figure 1). No significant inter-study heterogeneity was observed (I ^2 ^= 27.2%, P value for Heterogeneity [P_Het_] = 0.20). The distribution of the ORs from individual studies in relation to their respective standard deviations (the funnel plot) was symmetric and the Egger test suggested a low probability of small-study bias (p = 0.78).

**Figure 2:**
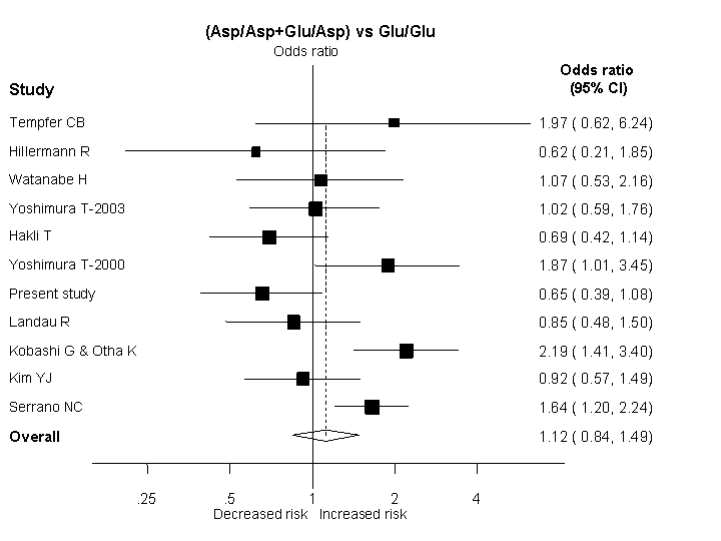
Results of published studies of association between the Glu298Asp polymorphism and pre-eclampsia. ORs for the outcome compared women carriers for the Asp298 allele vs women homozygous for the Glu298allele (Dominant model), sorted by study size.

**Figure 3:**
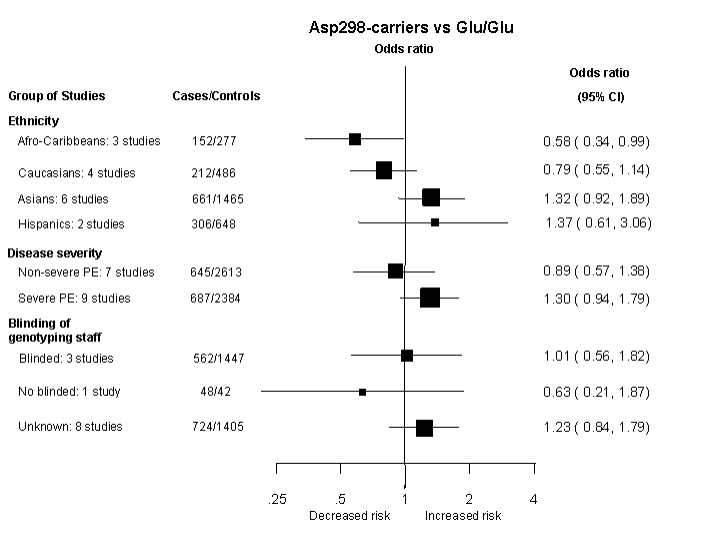
Sensitivity analysis of the Glu298Asp polymorphism and pre-eclampsia Summary odds ratio for pre-eclampsia for women carriers of the Asp298 allele compared to women homozygous for the Glu298 allele, divided by ethnic group, disease severity and blinding of the genotyping staff.

A dominant model indicated no increase of risk of pre-eclampsia for carriers of at least one Asp298 allele (OR: 1.12 [95%CI: 0.84–1.49]; p = 0.42) (Figure 2). There was evidence of substantial inter-study heterogeneity (I^2 ^= 65%; P_Het _= 0.001) with this model, partially explained by ethnicity (χ^2 ^= 17.17, 3 df, p = 0.001), while disease severity (severe vs non-severe pre-eclampsia; χ^2 ^= 1.85, 1 df, p = 0.17) and use of blinding of genotyping staff to the case-control status (χ^2 ^= 1.47, 2 df, p = 0.48) did not account for much of the heterogeneity (Figure 3). The distribution of the funnel plot was symmetric and the Egger test suggested a low probability of small-study bias (p = 0.35).

When an additive genetic model was evaluated the summary per allele OR of the Glu298Asp polymorphism for pre-eclampsia was 1.03 (95%CI: 0.79–1.34; p = 0.84). The funnel plot was symmetric and the Egger test suggested a low probability of small-study bias (p = 0.20).

## Conclusion

The main finding of the case-control study and the meta-analysis was that on evidence obtained to date, women homozygous for the Asp298 allele were not associated with a significantly increased risk of pre-eclampsia when compared with carriers of the Glu298 allele (summary OR: 1.28 [0.76–2.16]; p = 0.34). Similar results were obtained using other genetic models of inheritance. However, an exploratory analysis that must be treated with appropriate caution suggested that the effect of this candidate-gene might be different according to ethnic background.

The aetiology of pre-eclampsia remains complex. Identification of candidate-genes for pre-eclampsia could aid substantially in the understanding of this important public health problem and provide clues for the prevention and treatment. It might also have wide relevance since women with a history of pre-eclampsia are at increased risk of cardiovascular disease in later life [14]. Endothelial dysfunction due to reduced NO availability has been implicated as potentially important mechanism in both pre-eclampsia [33] and cardiovascular disease [34].

Pre-eclampsia has an inherited component and it is likely that many genes are involved [35]. Previous linkage studies in affected siblings pairs have implicated the eNOS gene locus on chromosome 7q35 to 36 [36,37]. The common Asp298 variant of the eNOS might be susceptible to enhanced proteolytic cleavage [38,39] and this could contribute to the abnormally low NO production and increased cardiovascular risk observed in carriers of this allele, [13,39] though these findings have been debated [40]. These results have been supported by human studies that have reported that healthy pregnant women carriers of the Glu298Asp polymorphism have impairment in the flow mediated dilation, an indirect tool to evaluate NO bioavailability [12]. Although, it has been suggested that carriers of this allele might be more susceptible to endothelial dysfunction and hence the subsequent development of pre-eclampsia [33], in the present report we failed to detect a significant positive association between the Glu298Asp polymorphism and pre-eclampsia.

The lack of evidence of increased pre-eclampsia risk for the eNOS Glu298Asp variant may be explained by: (i) the absence of a biological effect, (ii) failure to detect a small but important increase in susceptibility to pre-eclampsia because of inadequate statistical power or (iii) the presence of real genetic heterogeneity according to ethnic background (a significant genotype-disease association in some, but not all ethnic groups). It has been recently suggested, that for complex diseases with a low sibling recurrence risk such as pre-eclampsia, several candidate-genes with a small to moderate effect, rather than a few candidate-genes with a high effect on incidence of the disease, is the more likely model to explain such genetic susceptibility [11]. This suggestion has been recently supported, for findings in other complex disease like ischaemic stroke where in a systematic analysis of all candidate-genes evaluated thus far, just a few genes with a small to moderate effect (ORs between 1.2 and 1.45) were shown to exhibit a significant association with the disease[41].

In order to increase the statistical power, we therefore combined all available studies in a meta-analysis. In addition to our study, eleven other studies were identified, but only nine studies providing 3513 women (1129 cases & 2384 controls) under a genetic recessive model were included in our main comparison. A very large case-control study or a meta-analysis of smaller studies, including about 5600 Caucasian women would be needed to have an 80% of power at a significance level of 5% to detect an OR of 1.3 under a recessive model of inheritance. A sample size approximately three times higher would be needed to detect the same OR among non-Caucasian populations because of the lower allele frequency of Asp298. Thus, a small to moderate effect on incidence of pre-eclampsia for the Glu298Asp cannot be totally excluded on current evidence. Since recruitment of data sets of the required size may be difficult "*de novo*" for a single centre, an alternative approach, which has been suggested in the cardiovascular arena, involves the recruitment and genotyping of smaller numbers of patients and controls from many centres according to uniform selection criteria and outcome definitions, and submission of the data to a common web-based repository for on line continuously up-dated meta-analysis. Such an approach would also help to minimise publication bias, which is a potential concern with meta-analysis of published genetic association studies. However, an alternative approach would be to conduct a very large multi-centre case-control study of several thousand of cases, which should be less prone to bias, in order to confirm or refute the role of candidate gene-variants with small to moderate effects in pre-eclampsia [11].

In summary, currently available data from genetic association studies do not provide positive evidence for a role of the eNOS Glu298Asp polymorphism in pre-eclampsia. Very large studies, in both Caucasians and non-Caucasian subjects, are required before a role for this gene can be excluded, and the development of very large datasets is an important research priority for genetic studies in this area [42].

## Competing interests

The author(s) declare they have no competing interest.

## Authors' contributions

CY recruited study subjects, abstracted medical records, entered data and drafted the manuscript.

JP contributed to the study design, performed statistical analysis, interpretation of the results and preparation of the manuscript.

MS contributed to the study design and interpretation of the results.

MKS performed all the PCR tests.

KN provided clinical authorisation and support for the study.

AH conceived methodology and supervision of PCR tests, conceived and co-ordinated the study, reviewed the statistical analyses and preparation of the manuscript.

All authors have read and approved the final manuscript.

## Pre-publication history

The pre-publication history for this paper can be accessed here:



## References

[B1] Robson SC, Hunter S, Boys RJ, Dunlop W (1989). Serial study of factors influencing changes in cardiac output during human pregnancy. Am J Physiol.

[B2] Williams DJ, Vallance PJ, Neild GH, Spencer JA, Imms FJ (1997). Nitric oxide-mediated vasodilation in human pregnancy. Am J Physiol.

[B3] Hingorani AD (2003). Endothelial nitric oxide synthase polymorphisms and hypertension. Curr Hypertens Rep.

[B4] Seligman SP, Buyon JP, Clancy RM, Young BK, Abramson SB (1994). The role of nitric oxide in the pathogenesis of preeclampsia. Am J Obstet Gynecol.

[B5] Choi JW, Im MW, Pai SH (2002). Nitric oxide production increases during normal pregnancy and decreases in preeclampsia. Ann Clin Lab Sci.

[B6] Salonen Ros H, Lichtenstein P, Lipworth L, Cnattingius S (2000). Genetic effects on the liability of developing pre-eclampsia and gestational hypertension. Am J Med Genet.

[B7] Krotz S, Fajardo J, Ghandi S, Patel A, Keith LG (2002). Hypertensive disease in twin pregnancies: a review. Twin Res.

[B8] Lachmeijer AM, Arngrimsson R, Bastiaans EJ (2001). Mutations in the gene for methylenetetrahydrofolate reductase, homocysteine levels, and vitamins status in women with a history of pre-eclampsia. Am J Obstet Gynecol.

[B9] Morgan L, Crawshaw S, Baker PN (1999). Maternal and fetal angiotensinogen gene allele sharing in pre-eclampsia. Br J Obstet Gynaecol.

[B10] Kosmas IP, Tatsioni A, Ioannidis JP (2003). Association of Leiden mutation in factor V gene with hypertension in pregnancy and pre-eclampsia: a meta-analysis. J Hypertens.

[B11] Colhoun HM, McKeigue PM, Davey Smith G (2003). Problems of reporting genetic associations with complex outcomes. Lancet.

[B12] Savvidou MD, Vallance PJ, Nicolaides KH, Hingorani AD (2001). Endothelial nitric oxide synthase gene polymorphism and maternal vascular adaptation to pregnancy. Hypertension.

[B13] Casas JP, Bautista LE, Humphries SE, Hingorani AD (2004). Endothelial Nitric Oxide Synthase Genotype and Ischemic Heart Disease: Meta-Analysis of 26 Studies Involving 23028 Subjects. Circulation.

[B14] Smith GC, Pell JP, Walsh D (2001). Pregnancy complications and maternal risk of ischaemic heart disease: a retrospective cohort study of 129,290 births. Lancet.

[B15] Yoshimura T, Yoshimura M, Tabata A, Shimasaki Y, Nakayama M, Miyamoto Y, Saito Y, Nakao K, Yasue H, Okamura H (2000). Association of the missense Glu298Asp variant of the endothelial nitric oxide synthase gene with severe preeclampsia. J Soc Gynecol Investig.

[B16] Kobashi G, Yamada H, Ohta K, Kato E, Ebina Y, Fujimoto S (2001). Endothelial nitric oxide synthase gene (NOS3) variant and hypertension in pregnancy. Am J Med Genet.

[B17] Hakli T, Romppanen EL, Hiltunen M, Helisalmi S, Punnonen K, Heinonen S (2003). Endothelial nitric oxide synthase polymorphism in preeclampsia. J Soc Gynecol Investig.

[B18] Yoshimura T, Chowdhury FA, Yoshimura M, Okamura H (2003). Genetic and Environmental Contributions to Severe Preeclampsia: Lack of Association with the Endothelial Nitric Oxide Synthase Glu298Asp Variant in a Developing Country. Gynecol Obstet Invest.

[B19] Landau R, Xie HG, Dishy V, Wood AJ, Stein CM, Smiley RM (2004). No association of the Asp298 variant of the endothelial nitric oxide synthase gene with preeclampsia. Am J Hypertens.

[B20] Ohta K, Kobashi G, Hata A, Yamada H, Minakami H, Fujimoto S, Kondo K, Tamashiro H (2003). Association between a variant of the glutathione S-transferase P1 gene (GSTP1) and hypertension in pregnancy in Japanese: interaction with parity, age, and genetic factors. Semin Thromb Hemost.

[B21] Tempfer CB, Jirecek S, Katrin Riener E, Zeisler H, Denschlag D, Hefler L, Husslein PW (2004). Polymorphisms of thrombophilic and vasoactive genes and severe preeclampsia: a pilot study. J Soc Gynecol Investig.

[B22] Watanabe H, Hamada H, Yamakawa-Kobayashi K, Yoshikawa H, Arinami T (2001). Evidence for an association of the R485K polymorphism in the coagulation factor V gene with severe preeclampsia from screening 35 polymorphisms in 27 candidate genes. Thromb Haemost.

[B23] Serrano NC, Casas JP, Díaz LA, Páez C, Mesa CM, Cifuentes R, Monterrosa M, Bautista A, Hawe E, Hingorani AD, Vallance P, López-Jaramillo P (2004). Endothelial Nitric Oxide Synthase Genotype and Risk of Preeclampsia: a multi-centre case-control study. Hypertension.

[B24] Yu CK, Papageorghiou AT, Parra M, Palma Dias R, Nicolaides KH, Fetal Medicine Foundation Second Trimester Screening Group (2003). Randomized controlled trial using low-dose aspirin in the prevention of pre-eclampsia in women with abnormal uterine artery Doppler at 23 weeks' gestation. Ultrasound in Obstet Gynecol.

[B25] Papageorghiou AT, Yu CK, Bindra R, Pandis G, Nicolaides KH, Fetal Medicine Foundation Second Trimester Screening Group (2001). Multicenter screening for pre-eclampsia and fetal growth restriction by transvaginal uterine artery Doppler at 23 weeks of gestation. Ultrasound Obstet Gynecol.

[B26] Brown MA, Lindheimer MD, de Swiet M, Van Assche A, Moutquin JM (2001). The classification and diagnosis of the hypertensive disorders of pregnancy: statement from the International Society for the Study of Hypertension in Pregnancy (ISSHP). Hypertens Pregnancy.

[B27] Shadish WR, Haddock CK, Cooper H, Hedges LV (1994). Combining estimates of effect size. The handbook of Research Synthesis.

[B28] DerSimonian R, Laird NM (1986). Meta-analysis in clinical trials. Controlled Clinical Trials.

[B29] Deeks JJ, Higgins JPT, Altman DG, editors (1986). Analysing and presenting results. In: Alderson P, Green S, Higgins J, editors. Cochrane Reviewers' Handbook 4.2.2. http://www.cochrane.org/resources/handbook/hbook.htm.

[B30] Egger M, Smith GD, Schneider M, Minder C (1997). Bias in meta-analysis detected by a simple, graphical test. BMJ.

[B31] Hillermann R, Carelse K, Gebhardt GS (2005). The Glu298Asp variant of the endothelial nitric oxide synthase gene is associated with an increased risk for abruptio placentae in pre-eclampsia. J Hum Genet.

[B32] Kim YJ, Park HS, Lee HY, Ha EH, Suh SH, Oh SK, Yoo HS Reduced l-arginine level and decreased placental eNOS activity in preeclampsia. Placenta.

[B33] Savvidou MD, Hingorani AD, Tsikas D, Frolich JC, Vallance P, Nicolaides KH (2003). Endothelial dysfunction and raised plasma concentrations of asymmetric dimethylarginine in pregnant women who subsequently develop pre-eclampsia. Lancet.

[B34] Hingorani AD (2001). Polymorphisms in endothelial nitric oxide synthase and atherogenesis. Atherosclerosis.

[B35] Pridjian G, Puschett JB (2002). Preeclampsia: Part 2: experimental and genetic considerations. Obstet Gynecol Surv.

[B36] Guo G, Lade JA, Wilton AN, Moses EK, Grehan M, Fu Y, Qiu H, Cooper DW, Brennecke SP (1999). Genetic susceptibility to pre-eclampsia and chromosome 7q36. Hum Genet.

[B37] Lade JA, Moses EK, Guo G, Wilton AN, Grehan M, Cooper DW, Brennecke SP (1999). The eNOS gene: a candidate for the preeclampsia susceptibility locus?. Hypertens Pregnancy.

[B38] Tesauro M, Thompson WC, Rogliani P, Qi L, Chaudhary PP, Moss J (2000). Intracellular processing of endothelial nitric oxide synthase isoforms associated with differences in severity of cardiopulmonary diseases: cleavage of proteins with aspartate vs. glutamate at position 298. Proc Natl Acad Sci USA.

[B39] Persu A, Stoenoiu MS, Messiaen T, Davila S, Robino C, El-Khattabi O, Mourad M, Horie S, Feron O, Balligand JL, Wattiez R, Pirson Y, Chauveau D, Lens XM, Devuyst O (2002). Modifier effect of eNOS in autosomal dominant polycystic kidney disease. Hum Mol Genet.

[B40] Fairchild TA, Fulton D, Fontana JT, Gratton JP, McCabe TJ, Sessa WC (2001). Acidic hydrolysis as a mechanism for the cleavage of the Glu(298)-->Asp variant of human endothelial nitric-oxide synthase. J Biol Chem.

[B41] Casas JP, Hingorani AD, Bautista LE, Sharma P (2004). Meta-analysis of genetic studies in ischemic stroke: thirty-two genes involving approximately 18,000 cases and 58,000 controls. Arch Neurol.

[B42] Khoury MJ, Davis R, Gwinn M, Lindegren ML, Yoon P (2005). Do we need genomic research for the prevention of common diseases with environmental causes?. Am J Epidemiol.

